# Value of the Neuro-Otologic Tests in the Army from a Diagnostic Standpoint

**Published:** 1919

**Authors:** 


***Value of the Neuro-Otologic Tests in The Army from a Diagnostic Standpoint.*** By E. R. Carpenter. *Journal of the American Medical Association*, Sept., 14, 1918.

There are many borderline cases among the men already in the service, to whom the Barany tests and correct interpretation of them are highly applicable if we maintain the same standard for ear work that we have established for the other branches of medicine.

Owing to the anatomic relation of the vestibular end-organ to the organ of hearing in the labyrinth and the common nerve trunk for the two senses, as well as a close central relation, the co-operation of the otologist and neurologist will almost invariably lead to a correct diganosis ; at least they can determine whether the trouble is functional or organic. The soldier who complains of dizziness or of dizzy spells is entitled to just as much consideration as the one who complains of poor vision or deafness. Not all men troubled with dizziness necessarily have vestibular disease. However, the significance of the symptom is so marked that one should never overlook the possibility of a lesion in the structures constituting the vestibular apparatus.

The peripheral nerve ending may be involved by any disease common to the middle and internal ear. The nerve trunk is subject to various disturbances, and when we consider the extensive ramifications of the vestibular fibres in the spinal cord, the medulla, the cerebellum and the cerebral cortex, as well as the vestibular relation to all the cranial nerve nuclei, we can appreciate the opportunities for intracranial involvement.

Through the various connections of the cranial nerve nuclei, reflex irritation may occur and produce dizziness, as is frequently encountered in refraction work, nasal examinations, acute indigestion, etc., while toxemia from any source, as chronic indigestion, chemical absorption, infectious and constitutional diseases, may account for other cases.

This brings us to the conclusion that a more general use of the tests is indicated when dizziness is encountered, and that a closer co-operation of the otologist with the neurologist is necessary in this work.

